# Survival benefit of anlotinib in T790M‐positive non‐small‐cell lung cancer patients with acquired osimertinib resistance: A multicenter retrospective study and exploratory in vitro study

**DOI:** 10.1002/cam4.6232

**Published:** 2023-06-30

**Authors:** Ya Chen, Hongyu Liu, Nana Hu, Yanan Wang, Zhengyu Yang, Junqiang Zhang, Baohui Han

**Affiliations:** ^1^ Department of Respiratory and Critical Care Medicine, The First Affiliated Hospital of USTC, Division of Life Science and Medicine University of Science and Technology of China Hefei China; ^2^ Department of Pulmonary, Shanghai Chest Hospital Shanghai Jiao Tong University School of Medicine Shanghai China; ^3^ Department of Oncology The Affiliated Hospital of QingDao University QingDao China; ^4^ Department of Respiratory and Clinical Care Medicine Shanghai Jiao Tong University Affiliated Sixth People's Hospital Shanghai China

**Keywords:** anlotinib, non‐small‐cell lung cancer, osimertinib, resistance

## Abstract

**Objectives:**

Acquired resistance represents a bottleneck to epidermal growth factor receptor (EGFR) tyrosine kinase inhibitor (TKI) treatment in lung cancer. Our study aimed to explore the efficacy of antiangiogenic‐based therapy in osimertinib‐resistant NSCLC patients and assess the efficacy of anlotinib in vitro study.

**Methods:**

Our multicenter study retrospectively collected 268 osimertinib‐resistant NSCLC patients with EGFR T790M mutation and explored the efficacy of anlotinib in patients and in vitro.

**Results:**

PFS was significantly longer in the antiangiogenic‐based therapy group than in the immunotherapy group (HR: 0.71, *p* = 0.050) and the chemotherapy group (HR: 0.28, *p* = 0.001). Both the ORR and DCR of the antiangiogenic‐based group were higher than the immunotherapy group and the chemotherapy group. Subgroup analysis showed a trend of more benefits from the anlotinib‐based therapy than the bevacizumab‐based therapy in terms of PFS (HR: 0.63, *p* = 0.087) and OS (HR: 0.52, *p* = 0.063). In vitro assays verified that anlotinib alone or combined with osimertinib exerted potent cytotoxicity to T790M‐mutant H1975 cell line with acquired osimertinib resistance.

**Conclusions:**

Our study suggested that antiangiogenic‐based therapy might improve PFS and OS in EGFR‐mutant NSCLC patients with acquired resistance to osimertinib. Moreover, anlotinib‐based therapy could be a promising effective treatment for this group of patients.

## Graphical abstract

Our study suggested that antiangiogenic‐based therapy might improve PFS and OS in EGFR‐mutant NSCLC patients with acquired resistance to osimertinib. Moreover, we also conducted preliminary in vitro assays to evaluate the efficacy of Anlotinib and in combination with osimertinib in EGFR T790M‐mutant osimertinib‐resistant NSCLC cell line H1975OR.

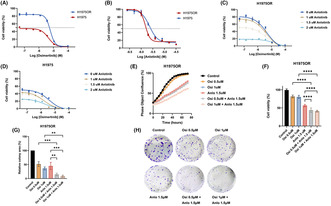

## 1 INTRODUCTION

Approximately 45.1% of non‐small‐cell lung cancer (NSCLC) Asian patients bear epidermal growth factor receptor (EGFR) mutation.^1^ Since the discovery of first‐generation EGFR‐tyrosine kinase inhibitor (TKI), targeted therapy has become a viable choice for NSCLC patients with EGFR mutation. Today, the third‐generation EGFR‐TKI Osimertinib has been proven to bring greater benefit to this group of patients as second‐line treatment after acquiring T790M mutation from previous EGFR‐TKI therapy or as first‐line treatment.^2^, ^3^ However, acquired resistance represents a bottleneck to osimertinib treatment in lung cancer. Due to the patient's genetic and clinical context complexity, the spectrum of resistance mechanisms is mostly heterogeneous and not well established, including EGFR‐dependent (such as C797S mutation) and EGFR‐independent mechanisms (such as MET amplification). Two main strategies under investigation were alternative TKIs or EGFR TKIs combined with other agents. For EGFR mutations after resistance, first or second‐generation TKIs or in combination with osimertinib were reported to be a potential way to overcome the resistance. New generation TKIs specifically targeted these resistant mutations, such as JBJ‐04‐125‐02 and BLU‐945, are also being developed.^4^, ^5^, ^6^, ^7^ For patients with specific EGFR‐independent resistance mechanisms, the benefits of EGFR TKIs combined with other targeted agents (such as MET inhibitors, HER2 inhibitors, and HER3 inhibitors) are being evaluated.^8^, ^9^, ^10^ Additionally, the benefits and safety of immunotherapy or in combination with chemotherapy and antiangiogenic therapy are also being investigated in this scenario. Overall, multiple strategies aiming at osimertinib resistance are still under exploration. And management of NSCLC patients with acquired resistance to osimertinib is still inconclusive.

In addition, re‐biopsy is faced with different aspects of limitations in the real world, making it difficult for physicians to understand the mechanisms underlying osimertinib resistance of patients. At our center, most patients resistant to Osimertinib regularly receive antiangiogenic‐based therapy, immunotherapy, chemotherapy, or immunotherapy combined with chemotherapy under the guidance of physician evaluation. For antiangiogenic‐based therapy, anlotinib and bevacizumab were two common choices. However, recent results from multiple clinical trials suggested that the combination of osimertinib and bevacizumab failed to improve the PFS of patients with EGFR‐mutant non‐squamous NSCLC as first‐line treatment as well as of patients previously treated with EGFR‐TKIs.^11^, ^12^, ^13^ Anlotinib is an oral multi‐targeted TKI that inhibits VEGFR (1–3), PDGFR‐α/β, FGFR (1–4), and c‐Kit.^14^, ^15^ It showed potent anti‐angiogenesis effects and antitumor effects in vitro and in vivo.^15^ Based on the results of a Phase 3 randomized clinical trial (ALTER 0303) conducted at our center, anlotinib was approved for advanced NSCLC in China as a third‐line or beyond treatment.^16^ Furthermore, subgroup analysis of ALTER 0303 trial showed that Anlotinib monotherapy yielded longer OS for patients with EGFR mutation.^17^

Therefore, the standard care for a broader range of patients with acquired resistance to Osimertinib needs to be discovered. In this study, we compared the efficacy of regular regimens patients received at our center and investigated the potential antitumor effect of anlotinib or in combination with osimertinib in EGFR‐T790M mutant osimertinib‐resistant NSCLC cell line.

## 2 MATERIALS AND METHODS

### 2.1 Patients

Two hundred sixty‐eight patients form the First Affiliated Hospital of USTC and Shanghai Chest Hospital met the following inclusion criteria in this multicenter study: (1) advanced NSCLC with secondary EGFR exon 20 T790M mutation, (2) received at least one line of chemotherapy and osimertinib therapy, (3) received antiangiogenic therapy, immunotherapy, or chemotherapy after osimertinib resistance, (4) Eastern Cooperative Oncology Group performance status (ECOG PS) 0–1. The therapeutic schedule was decided by the physician under the principle that patients at high risk of bleeding should not be treated with antiangiogenic drugs, and patients with severe adverse effects to previous chemotherapy should not choose chemotherapy as a priority. This study was approved by the Ethics Committee of the Shanghai Chest Hospital (NO.KP17‐20) and The First Affiliated Hospital of USTC (202304062238000354629). The need for informed consent was waived due to the retrospective nature of the study.

### 2.2 Molecular analysis and clinical evaluation

EGFR detection was performed by next‐generation sequencing (NGS) or amplification refractory mutation system (ARMS). The disease stage was decided on the 8th edition of the American Joint Committee on Cancer International Association for the Study of Lung Cancer (AJCC) (IASLC) tumor‐node‐metastasis (TNM) classification. Enhanced chest computed tomography (CT) and abdominal ultrasound scans were performed every 4–6 weeks or when symptoms progressed significantly. Enhanced brain magnetic resonance imaging (MRI) was examined every 4–6 months if there was no lesion at baseline and no symptoms thereafter.

### 2.3 Cell line and reagents

Human lung adenocarcinoma osimertinib‐resistant cell line H1975OR was generously provided by the department of pharmacology, school of medicine, Shanghai Jiaotong University. Cells were cultured in RPMI‐1640 culture medium (Gibco; Thermo Fisher Scientific, Inc.) added with 10% fetal bovine serum (Gemini Bio) at 37 °C with 5% of CO_2_.

Osimertinib was purchased from Selleck Chemical LLC. Anlotinib was generously offered by Chia Tai Tianqing Pharmaceutical Group Co., Ltd (Nanjing, China). Osimertinib and anlotinib were dissolved with DMSO (Sigma) and stored at −20°C until use. CCK‐8 assay (Proteintech) was utilized to determine cell viability.

### 2.4 Cell growth and viability assay

Briefly, cells were seeded 4000 per well in 96‐well plates and treated with indicated agents. For cell growth assay, plates were placed in Incucyte® ZOOM Live‐Cell Analysis System (Essen BioScience) for 3 days, and phase object confluence was calculated by analysis software under the guidance of the manual. For cell viability assay, after being cultured with indicated agents for 3 days, cells were treated with CCK‐8 reagents, and the optical density at 450 nm was measured with a plate reader according to the manufacturer's instructions.

### 2.5 Colony formation assay

Cells were seeded 4000 per well in six‐well plates and treated with indicated agents. After being cultured for 7 days, cells were fixed with 4% paraformaldehyde fix solution (Biosharp) for 30 min and stained with crystal violet staining solution (Beyotime) overnight. The colony area was determined using Fiji ImageJ software.^18^

### 2.6 Statistical analysis

The categorical variables were compared by chi‐square test. The endpoints were PFS (calculated from the start of antiangiogenic‐based therapy, immunotherapy, or chemotherapy that after osimertinib resistance to disease progression); OS (from the start of antiangiogenic‐based therapy, immunotherapy, or chemotherapy to death or the last follow‐up) and ORR (the ratio of complete and partial response). Hazard ratios (HR) and 95% confidence intervals were performed by Cox proportional hazards regression model, and the survival difference was visualized by Kaplan–Meier curve and log‐rank test. To avoid the influence of confounding factors, factors with p values less than 0.1 in univariate analysis were included in multivariate analysis. To reduce the selection bias as much as possible, propensity score matching (PSM) method was applied to anlotinib‐based and bevacizumab‐based group patients at a 1:1 ratio via nearest‐neighbor method. Propensity scores for included patients were calculated by multiple logistic regression with adjustment for clinical characteristics, including age, sex, smoking history, surgery history, number of metastatic lesions, brain metastasis, and EGFR mutation subtype. A total of 42 pairs were successfully matched.

Quantitative data from in vitro assays were shown as mean ± SD. The statistical difference between groups was determined using the Mann–Whitney test, chi‐square test, or Kruskal–Wallis test. All statistical analyses were performed using SPSS version 22.0 (IBM Corporation) and GraphPad Prism 9.

## 3 RESULTS

### 3.1 Clinical features

Two hundred sixty‐eight patients who met the eligibility criteria were included in this study. The clinicopathologic characteristics of eligible patients are shown in Table 1. Most patients were female (54.5%) and non‐smoker (71.6%). 64 patients (23.9%) had brain metastasis. The most common EGFR mutation type was EGFR^*L858R/T790M*^ (57.1%). Most patients (*N* = 145) received antiangiogenic‐based therapy, including 103 anlotinib‐treated patients and 42 bevacizumab‐treated patients. 47 received anti‐PD‐1 therapy or anti‐PD‐1 therapy combined with chemotherapy. 76 patients received chemotherapy alone.

**TABLE 1:** Clinicopathological characteristics of 268 EGFR‐mutant patients.

Characteristics	Number	Percent(%)
Age(median age, Range)	61 (36–91)	–
≥65	88	32.8
<65	180	67.2
Sex		
Male	122	45.5
Female	146	54.5
Smoking history		
Yes	76	28.4
No	192	71.6
Recurrence after surgery		
Yes	117	43.7
No	151	56.3
Number of metastatic lesions		
1–3	198	73.9
4–5	70	26.1
Brain metastasis		
Yes	64	23.9
No	204	76.1
EGFR mutation subtype		
19del	115	42.9
21L858R	153	57.1
Treatment		
Angio	145	54.1
Bevacizumab‐based treatment	42	29.0
Bevacizumab	20	
Bevacizumab combined with osimertinib	22	
Anlotinib‐based treatment	103	71.0
Anlotinib	69	67.0
Anlotinib combined with osimertinib	34	33.0
Immuno	47	
PD‐1	33	70.2
PD‐1 + chemo	14	29.8
Chemo	76	28.4

### 3.2 Progression‐free survival (PFS) and overall survival (OS)

**FIGURE 1:**
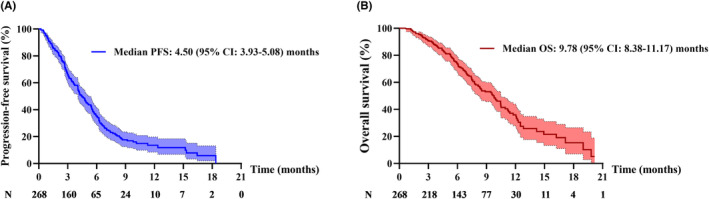
(A) Progression‐free survival and (B) overall survival of patients with acquired osimertinib resistance NSCLC in the overall population. 95% confidence intervals are shown in light colors.

During the median follow‐up of 9.63 months (range: 0.63–20.17) for all the 268 patients, 190 (70.1%) patients developed PD. The overall median PFS was 4.50 months (95% CI: 3.93–5.08) (Figure 1A). The univariate analysis results showed that brain metastasis (*p* = 0.072) and treatment [*p* (antiangiogenic‐based therapy vs. chemotherapy) <0.001, *p* (immunotherapy vs. chemotherapy) <0.001)] were associated with PFS (Table 2). Furthermore, the multivariate analysis found that patients who received antiangiogenic‐based therapy or immunotherapy had longer PFS than those who received chemotherapy. Patients without brain metastasis had longer PFS than those with brain metastasis (Table 2). These results indicated that brain metastasis and treatment were independent prognostic factors of PFS.

**TABLE 2:** Univariable and multivariable analysis of covariables associated with PFS and OS.

Characteristics	Category	Progression‐free survival (*n* = 231)				Overall survival (*n* = 231)	
		Univariate analysis		Multivariate analysis		Univariate analysis	
		Hazard ratio	*p*	Hazard ratio	*p*	Hazard ratio	*p*
Age	<65 versus >65 years	1.13 (0.82–1.55)	0.452			0.78 (0.54–1.13)	0.183
Sex	Male versus female	1 (0.86–1.15)	0.976			1.09 (0.77–1.54)	0.647
Smoking history	Yes versus no	1.14 (0.83–1.57)	0.432			1.19 (0.81–1.76)	0.372
Recurrence after surgery	yes versus no	0.95 (0.71–1.26)	0.708			1.05 (0.74–1.49)	0.796
Number of metastatic lesions	1–3 versus 4–5	1.33 (0.94–1.88)	0.103			0.99 (0.65–1.51)	0.976
Brain metastasis	Yes versus no	1.35 (0.97–1.86)	0.072	1.39 (1–1.92)	0.049	1.21 (0.82–1.77)	0.332
EGFR mutation subtype	19del versus 21L858R	1.03 (0.77–1.38)	0.833			1.03 (0.73–1.46)	0.882
Treatment	Angio versus chemo	0.28 (0.2–0.39)	<0.001	0.28 (0.2–0.39)	<0.001	0.14 (0.09–0.21)	<0.001
	Immuno versus chemo	0.44 (0.28–0.67)	<0.001	0.44 (0.29–0.69)	<0.001	0.23 (0.13–0.38)	<0.001

Death occurred in 128 (47.8%) patients in the overall population. The median OS was 9.78 (95% CI: 8.38–11.17) (Figure 1B). Univariate analysis also found that treatment was associated with OS (Table 2), and patients in the antiangiogenic‐based therapy group had longer OS [*p* (antiangiogenic‐based therapy vs. chemotherapy) <0.001, *p* (immunotherapy vs. chemotherapy) <0.001)], indicating that treatment was an independent prognostic factor of OS.

### 3.3 Survival analysis of treatment groups

**FIGURE 2:**
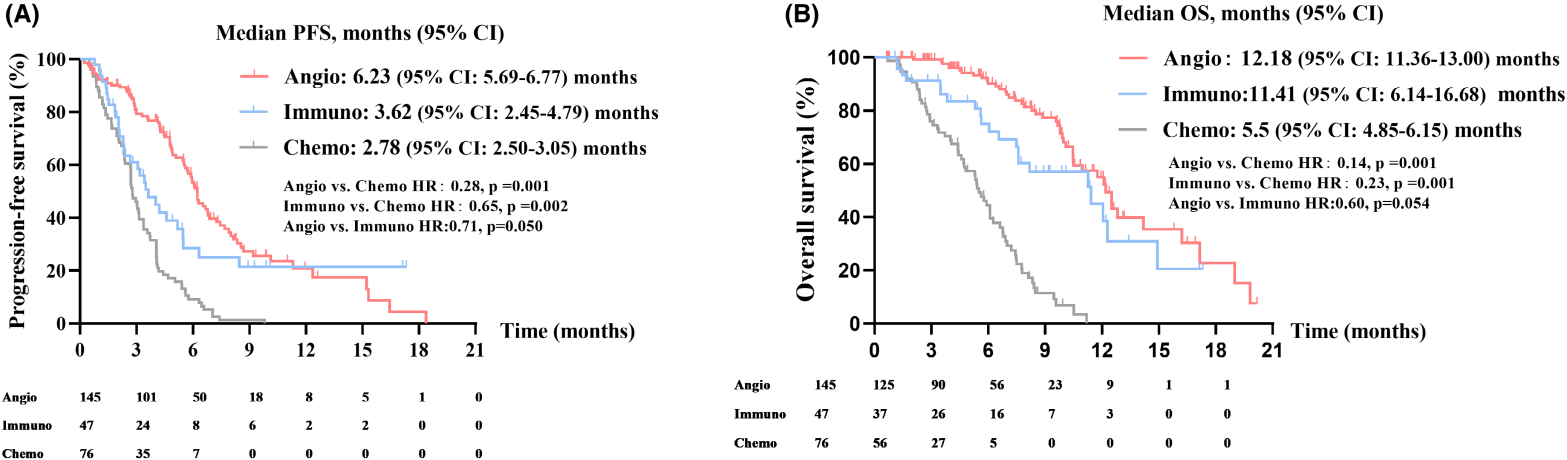
Kaplan–Meier analysis of (A) progression‐free survival and (B) overall survival of patients with acquired osimertinib resistance NSCLC treated with antiangiogenic‐based therapy (Angio), immunotherapy (Immuno) or chemotherapy (Chemo).

**FIGURE 3:**
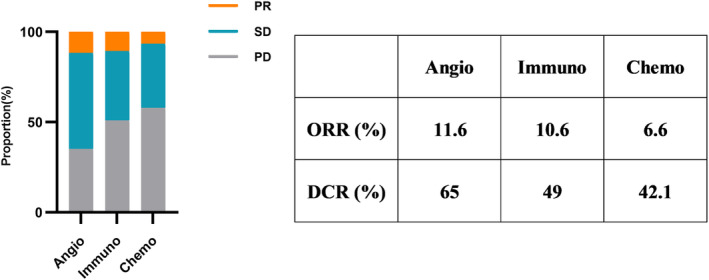
The ORR and DCR of patients in the antiangiogenic‐based therapy group (Angio), immunotherapy group (Immuno) and chemotherapy group (Chemo).

To further explore the difference in survival among the three regimens, we divided the overall population into three groups based on their treatment and analyzed the PFS and OS, respectively. The clinicopathologic characteristics of the three groups are shown in Table 3. There were no significant differences among the three groups in terms of clinicopathologic characteristics, suggesting that there was no significant selection bias. The median PFS was 6.23 (95% CI: 5.69–6.77) months in the antiangiogenic‐based therapy group, 3.62 (95% CI: 2.45–4.79) months in the immunotherapy group, and 2.78 (95% CI: 2.50–3.05) months in the chemotherapy group (Figure 2A). PFS was significantly longer in the antiangiogenic‐based therapy group than in the immunotherapy group (HR:0.71, *p* = 0.050) and the chemotherapy group (HR:0.28, *p* = 0.001). Meanwhile, the median OS was 12.18 (95% CI: 11.36–13.00) months in the antiangiogenic‐based therapy group, 11.41 (95% CI: 6.14–16.68) months in the immunotherapy group, and 5.5 (95% CI: 4.85–6.15) months in the chemotherapy group (Figure 2B). And OS was significantly longer in the antiangiogenic‐based therapy group than in the chemotherapy group (HR:0.14, *p* = 0.001), while there was no significant difference between the antiangiogenic‐based therapy group and the immunotherapy group (HR:0.60, *p* = 0.054). We also compared the overall response rate (ORR) and disease control rate (DCR) of the three groups. Results showed that both the ORR and DCR of the antiangiogenic‐based group were longer than the immunotherapy group and the chemotherapy group (Figure 3. ORR: 11.6% vs. 10.6% and 6.6%; DCR: 65% vs. 49% and 42.1%). Overall, these findings indicated that antiangiogenic‐based therapy could be a beneficial choice for NSCLC patients resistant to osimertinib as a fourth‐ or later‐line treatment.

**TABLE 3:** Clinicopathological characteristics of 268 EGFR‐mutant patients treated with different regiments.

Characteristics	Angio (*N* = 145)	Immuno (*N* = 47)	Chemo (*N* = 76)	*p* Value
Age				
≥65	49	19	20	0.252
<65	96	28	56	
Sex				
Male	61	25	36	0.384
Female	84	22	40	
Smoking history				
Yes	35	18	23	0.158
No	110	29	53	
Recurrence after surgery				
Yes	61	20	36	0.742
No	84	27	40	
Number of metastatic lesions				
1–3	106	39	53	0.254
4–5	39	8	23	
Brain metastasis				
Yes	38	11	15	0.561
No	107	36	61	
EGFR mutation subtype				
19del	61	20	34	0.929
21L858R	84	27	42	

### 3.4 Survival analysis of antiangiogenic‐based therapy subgroups

**FIGURE 4:**
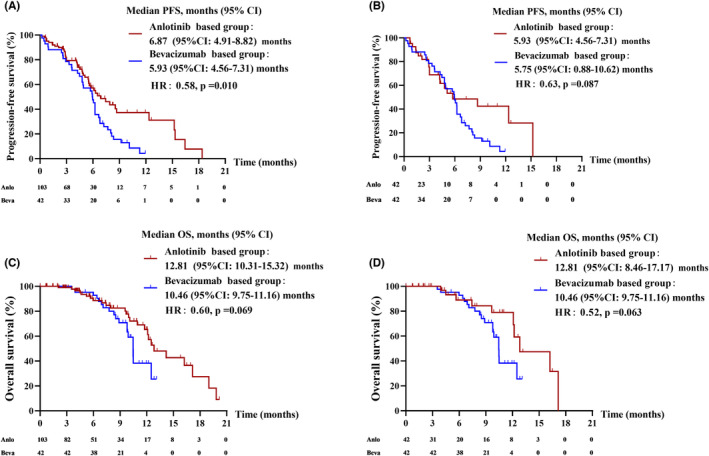
Kaplan–Meier analysis of progression‐free survival and overall survival of antiangiogenic therapy subgroups: (A) PFS before PS matching, (B) PFS after PS matching, (C) OS before PS matching and (D) OS after PS matching.

Next, we analyzed the difference between two antiangiogenic regimens—anlotinib and bevacizumab. To minimize the selection bias, we performed PS matching to the two subgroups. Clinicopathologic characteristics before and after PS matching are shown in Table 4. Before PS matching, the median PFS was 6.87 (95%CI: 4.91–8.82) months in the anlotinib‐based group and 5.93 (95% CI: 4.56–7.31) months in the bevacizumab‐based group (Figure 4A). PFS was significantly longer in the anlotinib‐based group than in the bevacizumab‐based group (HR:0.58, *p* = 0.010). The median OS was 12.81 (95% CI: 10.31–15.32) months in the anlotinib‐based group and 10.46 (95% CI: 9.75–11.16) months in the bevacizumab‐based group (Figure 4C). There was no significant difference between the two groups in terms of OS (HR:0.60, *p* = 0.069), but the survival curve showed a trend of more benefits from the anlotinib‐based group. After PS matching, the median PFS was 5.93 (95% CI: 4.56–7.31) months in the anlotinib‐based group and 5.75 (95% CI: 0.88–10.62) months in the bevacizumab‐based group (Figure 4B). The median OS was 12.81 (95% CI: 8.46–17.17) months in the anlotinib‐based group and 10.46 (95% CI: 9.75–11.16) months in the bevacizumab‐based group (Figure 4D). There was no significant difference between the two groups in terms of PFS and OS (PFS, HR: 0.63, *p* = 0.087; OS, HR:0.52, *p* = 0.063), and the survival curve also showed a trend of more benefits from the anlotinib‐based group. Results above suggested that NSCLC patients resistant to osimertinib might benefit more from anlotinib as a later‐line treatment.

**TABLE 4:** Clinicopathological characteristics of patients treated with antiangiogenic therapy before and after PS matching.

Characteristics	Before PS matching			After PS matching		
	Anlo (*N* = 103)	Bev (*N* = 42)	*p* Value	Anlo (*N* = 42)	Bev (*N* = 42)	*p* Value
Age						
≥65	41	8	0.017	10	8	0.595
<65	62	34		32	34	
Sex						
Male	48	13	0.083	13	13	1.000
Female	55	29		29	29	
Smoking history						
Yes	30	5	0.028	8	5	0.365
No	73	37		34	37	
Recurrence after surgery						
Yes	45	16	0.536	11	16	0.243
No	58	26		31	26	
Number of metastatic lesions						
1–3	72	34	0.173	34	34	1.000
4–5	31	8		8	8	
Brain metastasis						
Yes	23	15	0.096	13	15	0.643
No	80	27		29	27	
EGFR mutation subtype						
19del	48	13	0.083	12	13	0.811
21L858R	55	29		30	29	

### 3.5 Cell growth, viability, and colony formation assay

**FIGURE 5:**
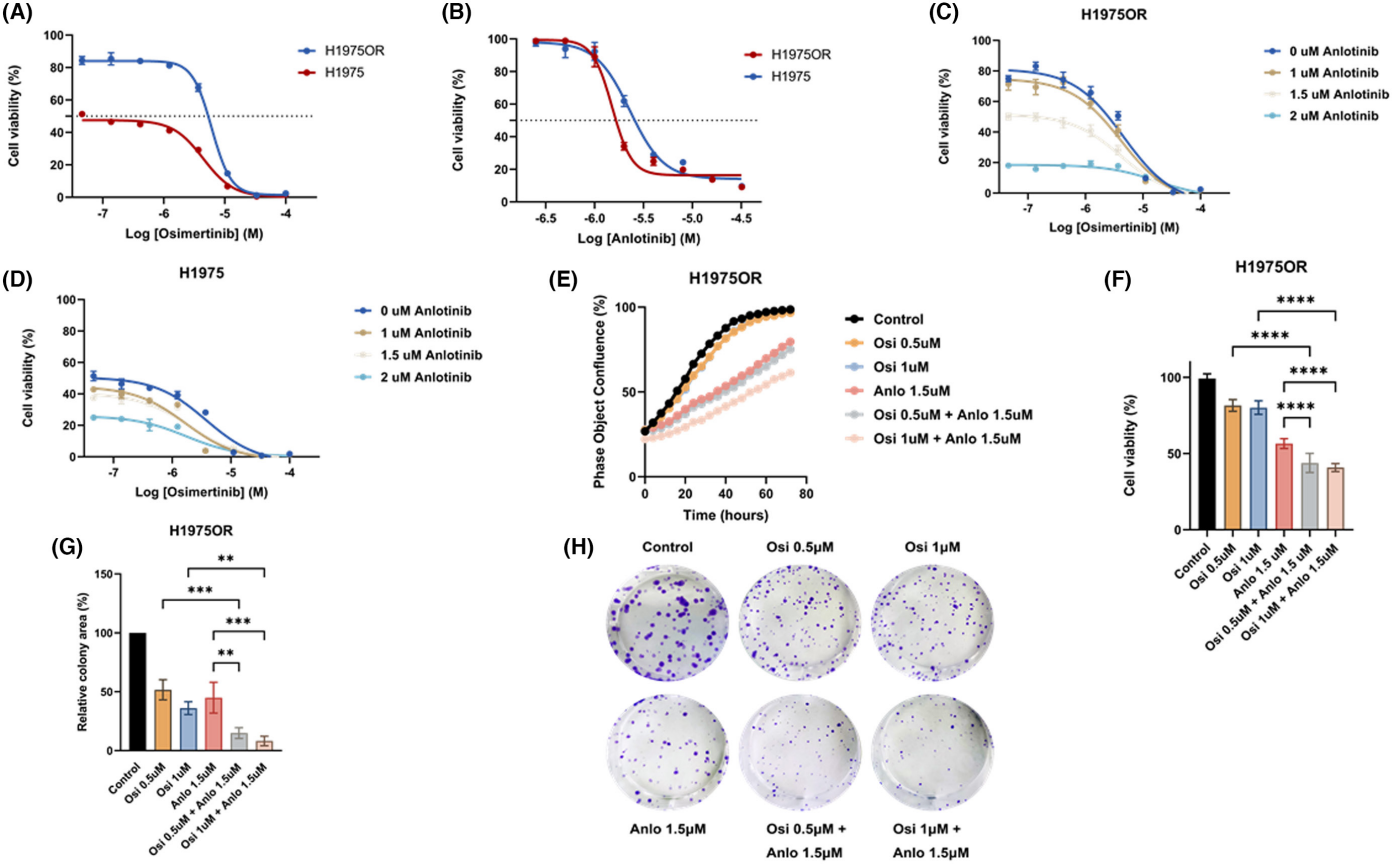
Cell viability assay of H1975OR cell line treated with osimertinib or anlotinib for 3 days(A, B). Cell viability assay of H1975OR and H1975 cell line treated with anlotinib and/or osimertinib at indicated concentrations for 3 days (C, D). Cell growth and viability of H1975OR cell line treated with anlotinib and/or osimertinib at indicated concentrations for 3 days (E, F). Colony formation assay of H1975OR cell line treated with anlotinib and/or osimertinib for 7 days (G, H). **p ≤ 0.01; ***p ≤ 0.001; ****p ≤ 0.0001.

To investigate the potential antitumor effect of anlotinib and in combination with osimertinib in osimertinib‐resistant EGFR‐T790M mutant NSCLC, we performed cell growth and viability assay in H1975 and osimertinib‐resistant H1975 cell line (H1975OR) treated with anlotinib and/or osimertinib. Our results confirmed that H1975OR cell line was resistant to osimertinib compared to parental cell line (H1975OR IC_50_ = 6.113 μM, Figure 5A). On the other hand, anlotinib alone yielded potent cytotoxic effects against H1975OR, which is also relatively more sensitive to it compared to parental cell line. (H1975OR IC_50_ = 1.513 μM; H1975 IC_50_ = 2.300 μM, Figure 5B). Next, we explored the effect of anlotinib combined with osimertinib. Cell lines were treated with the two agents at the indicated concentrations and applied to CCK‐8 assay. Results showed that the combination was more effective against H1975OR compared with the single agent of osimertinib (Figure 5C). While in parental cell line, osimertinib alone showed significant cytotoxicity and the combination did not yield sufficient cell viability inhibition as much as in H1975OR (Figure 5D). Furthermore, we cultured H1975OR cells treated with certain concentrations of anlotinib and/or osimertinib and compared the growth ability and viability among treatment groups. Our findings suggested that anlotinib was more potent than osimertinib and anlotinib combined with osimertinib showed greater inhibitory effects than anlotinib or osimertinib alone against H1975OR cell line (Figure 5E,F). We then employed colony formation assay to explore whether anlotinib or in combination with osimertinib inhibited cell growth in the long term. After being treated with the single agent or the combination, H1975OR cells were cultured for 7 days. Colony formation assay indicated that anlotinib combined with osimertinib had long‐term cytotoxic effects against H1975OR cell line (Figure 5G,H). These findings suggested that anlotinib and anlotinib combined with osimertinib could be a potentially effective regiment for osimertinib‐resistant EGFR‐T790M mutant NSCLC.

## 4 DISCUSSION

Our multicenter study retrospectively assessed the efficacy of three common treatments for patients with acquired osimertinib resistance at our center. Our findings suggested that this group of patients could benefit more from antiangiogenic‐based therapy than immunotherapy or chemotherapy. Additionally, anlotinib, a multi‐targeted TKI, single agent, or in combination with osimertinib, might yield more clinical benefits than bevacizumab alone or combined with osimertinib as a later‐line treatment after osimertinib resistance.

As mentioned above, the WJOG9717L trial firstly investigated whether osimertinib plus bevacizumab was better than osimertinib alone for untreated EGFR‐mutant NSCLC patients. Results showed no significant difference between the two groups in terms of PFS (mPFS: 22.1 months for osimertinib plus bevacizumab group and 20.2 months for osimertinib group, HR: 0.862 [95% CI: 0.531–1.397], *p* = 0.213).^11^ Meanwhile, the BOOSTER trial and WJOG8715L trial studied the efficacy of this combination for patients with confirmed EGFR‐T790M mutant NSCLC as second‐line treatment. The BOOSTER trial showed that there was no difference in mPFS between osimertinib plus bevacizumab group (15.4 months [95% CI 9.2–18.0 months]) and osimertinib group (12.3 months [95% CI 6.2–17.2 months]), (*p* = 0.83, HR: 0.96 [95% CI 0.68–1.37]). And the WJOG8715 trial also showed that compared with osimertinib alone, mPFS was not longer with osimertinib plus Bevacizumab (9.4 months vs. 13.5 months, HR: 1.44 [80% CI 1.00–2.08], *p* = 0.20).^12^, ^13^ However, the potential clinical effects of antiangiogenic therapy for patients with acquired osimertinib resistance remain unclarified. Data from our study suggested that antiangiogenic‐based therapy was potentially the best regimen compared with immunotherapy and chemotherapy. And subgroup analysis found that PFS (5.93 [95% CI: 4.56–7.31] months versus 5.75 [95% CI: 0.88–10.62] months) and OS (12.81 [95% CI: 8.46–17.17] months versus 10.46 [95%CI: 9.75–11.16] months) of the anlotinib‐based group were longer than the bevacizumab‐based group. However, there was no significant difference between the two groups after PS matching, and the insufficient sample size could be one of the reasons. Anlotinib and bevacizumab are both antiangiogenic medicine but showed different efficacy, which is possibly due to the different targets of the medicine. Anlotinib is a multi‐targeted TKI that inhibits VEGFR (1–3), PDGFR‐α/β, FGFR (1–4), and c‐Kit while bevacizumab is a monoclonal antibody that only targets the VEGF‐A. Further research is needed to prove our hypothesis.

On the other hand, immunotherapy is also under investigation in the setting of post‐osimertinib treatment. Previously study suggested that the efficacy of immunotherapy as monotherapy decreased for patients harboring EGFR mutations.^19^, ^20^ Recent studies such as KEYNOTE 789 trial (NCT03515837) focus more on immunotherapy combined with chemotherapy, results of which should be able to elucidate the role of immunotherapy for patients resistant to osimertinib. Recently, a Phase 3 trial, focusing on the efficacy of immunotherapy plus antiangiogenic therapy and chemotherapy for NSCLC patients who had progressed disease after EGFR‐TKI treatment, showed that mPFS was significantly longer in the sintilimab plus IBI305 plus chemotherapy group compared with chemotherapy alone (6.9 months [95% CI: 6.0–9.3] versus 4.3 months [95% CI: 4.1–5.4]; HR: 0.46 [95% CI: 0.34–0.64], *p* < 0.0001).^21^ Interestingly, though studies are incomparable due to the difference of baseline characteristics and other backgrounds, the similar mPFS of our study and this study (6.87 months vs. 6.9 months) might suggest that Anlotinib plus Osimertinib could be considered for this group of patients with immunotherapy or chemotherapy intolerance.

Based on the findings from our retrospective study, we also conducted preliminary in vitro assays to evaluate the efficacy of anlotinib and in combination with osimertinib in EGFR T790M‐mutant osimertinib‐resistant NSCLC cell line H1975OR. And our results indicated that anlotinib alone or combined with osimertinib showed potent cytotoxicity against H1975OR, providing a basis for further preclinical and clinical investigations.

However, this study had several limitations. First, this was a retrospective, non‐randomized study that selection bias existed inevitable due to unavoidable missing data. However, the baseline clinical characteristics of patients in the different groups were balanced well or were processed by PS matching, indicating that no large selection bias existed. Secondly, antiangiogenic‐based combination therapy in our study achieved a better efficacy while adverse events increased unavoidable. Due to the missing data in our retrospective study, adverse events and their impact on the patients' quality of life were not assessed. Clinical trials are needed to further explore the adverse effects and the efficacy. Thirdly, our study lacked in vivo experiments to further verify the efficacy of anlotinib alone or combined with osimertinib. Finally, more and more EGFR‐mutated, advanced NSCLC patients receive osimertinib as first‐line treatment, whether the antiangiogenic‐based therapy is also applicable to first‐line osimertinib resistant patients remains unclear, which needs to be further explored.

Collectively, our study showed that anlotinib alone or combined with osimertinib might be an effective treatment for patients with acquired osimertinib‐resistant EGFR T790M mutant NSCLC.

## AUTHOR CONTRIBUTIONS

**Ya Chen:** Conceptualization (equal); data curation (equal); writing – original draft (equal). **Hongyu Liu:** Conceptualization (equal); writing – review and editing (equal). **Nana Hu:** Methodology (equal); validation (equal). **Yanan Wang:** Formal analysis (equal); investigation (equal). **Zhengyu Yang:** Supervision (equal); validation (equal). **Junqiang Zhang:** Writing – review and editing (equal). **Baohui Han:** Writing – review and editing (equal).

## FUNDING INFORMATION

This research received funding from Science and Technology Commission of Shanghai Municipality (20YF1428100) and National Multi‐disciplinary Treatment Project for Major Diseases (2020NMDTP).

## CONFLICT OF INTEREST STATEMENT

The authors declares no conflict of interest.

## ETHICS STATEMENT

All procedures followed were in accordance with the ethical standards of the responsible committee on human experimentation (institutional and national) and with the Helsinki Declaration of 1975, as revised in 2000 (5). Informed consent was obtained from all patients for being included in the study. If the research was not conducted in accordance with the Helsinki Declaration, the authors must explain the rationale for their approach, and demonstrate that the institutional review body explicitly approved the doubtful aspects of the study.

## Data Availability

The data that support the findings of this study are available from the corresponding author upon reasonable request.
